# The Life Mission Theory II. The Structure of the Life Purpose and the Ego

**DOI:** 10.1100/tsw.2003.114

**Published:** 2003-12-11

**Authors:** Soren Ventegodt, Niels Jorgen Andersen, Joav Merrick

**Affiliations:** The Quality of Life Research Center, Copenhagen K, Denmark

**Keywords:** quality of life, QOL, philosophy, human development, holistic medicine, public health, salutogenesis, sense of coherence, personal development, ego, higher self, Denmark

## Abstract

Pursuing your life mission is often very difficult, and many frustrations are experienced along the way. Major failures to bring out our potential can cause us considerable emotional pain. When this pain is unbearable, we are induced to shift from one intention and talent to another that better allows us to adapt and survive. Thus, we become set on a course that brings out a secondary or tertiary talent instead of the primary talent. This talent displacement may be expressed as a loss of our true nature or true self. The new purpose in life now functions as the core of a new personality: the ego. The ego has a structure similar to that of the true self. It is anchored in a talent and it draws on subtalents. But the person who is centered in his or her ego is not as powerful or talented as the person he or she originally was, living the primary purpose of life. This is because the original personality (the true self or “higher self”) is still there, active and alive, behind the ego. Symptoms, disorders, and diseases may be explained by the loss of energy, joy in life, and intuitive competence because of inner conflicts, which may be alleviated or cured in the salutogenetic process of Antonovsky that helps patients find their sense of coherence or their primary purpose in life. Many cases of reduced ability to function, physically as well as psychologically, socially or sexually, can also be explained and alleviated in this way. When a person discovers his true talent and begins to use it with dedication, privately as well as professionally, his life will flourish and he may overcome even serious disease and great adversity in life. The salutogenetic process can also be called personal development or “quality of life as medicine”. It is important to note that the plan for personal development laid out by this theory is a plan not for the elimination of the ego, but for its cultivation. An existentially sound person still has a mental ego of course, but it is centered on the optimal verbal expression of the life mission. Such an ego is not in conflict with one's true self, but supports the life and wholeness of the person, although in an invisible and seamless way. The more developed the person, the more talents are taken into use. So although the core of existence remains the same throughout life, the healthy person continues to grow. As the number of talents we can call on is unlimited, the journey ends only at death. Understanding the concept of the ego, it is very easy for the physician to motivate the patient to go through a lot of difficulties in order to grow and develop, and when the patient fully understands the concept of the ego and the true self (higher self), the patient gets a strong feeling of direction in personal development, and a motivation to fight the internal obstacles for quality of life, health, and the ability to function.

## INTRODUCTION

The life mission theory claims that the core of human existence is to have a purpose in life, a "life mission" that can be articulated into words[1]. With some practice, a person may be able to capture his or her life mission in one single, short sentence, like "I create" or "I bring joy". The life mission can be seen variously as your purpose in life, your existential duty, gift to the world, true nature, greatest joy, path in life, key intention, or the realization of your greatest talent.

Let us explore the idea that the life mission is associated with a talent for doing what is implied by it. It is obvious that a life mission like "I create" presupposes a talent for creativity. To exploit such a talent, a person must draw on a number of secondary or auxiliary talents, such as talents for understanding, exploring, or connecting, which again draw on tertiary talents. For example, to support the talent for understanding, we must call on talents for analyzing, confronting, sensing, or intuition.

It seems very likely that every human being is capable of every possible intention or mission and so also possesses all the talents in the world. But we may suppose that human nature is structured such that only a few talents are in the foreground (maybe only one), while many are in the background, and some are in between. Using this core talent is what gives you the greatest pleasure possible in your life. To bring out your full potential in the Maslow'ian sense[2], you must develop not only your core talent, but also the secondary, tertiary, and other auxiliary talents supporting it.

In the best of worlds, we each find our life mission and core talent and manage to call on its auxiliary talents, so that they remain subservient to our life mission, our key intention or purpose in life. All too often, however, some auxiliary talent takes over and replaces the core talent. This displacement may be assumed to be pathogenic. Antonovsky[3], for example, describes it as a loss of coherence and Frankl as a loss of meaning[4].

## THE RISE AND THE STRUCTURE OF THE EGO

Pursuing your life mission is often very difficult, and many frustrations are experienced along the way. Major failures to bring out our potential can cause us considerable emotional pain. When this pain is unbearable, we are induced to shift from one intention and talent to another that better allows us to adapt and survive, given the circumstances in which we find ourselves. Thus, we become set on a course that brings out a secondary or tertiary talent, instead of the primary talent.

This talent displacement may be expressed as a loss of your true nature or true self. The new purpose in life now functions as the core of a new personality: the ego. The ego has a structure in a way similar to that of the true self. It is anchored in a talent and it draws on subtalents (Fig. 1). But the person who is centered in his or her ego is not as powerful or talented as the person he or she originally was, living the

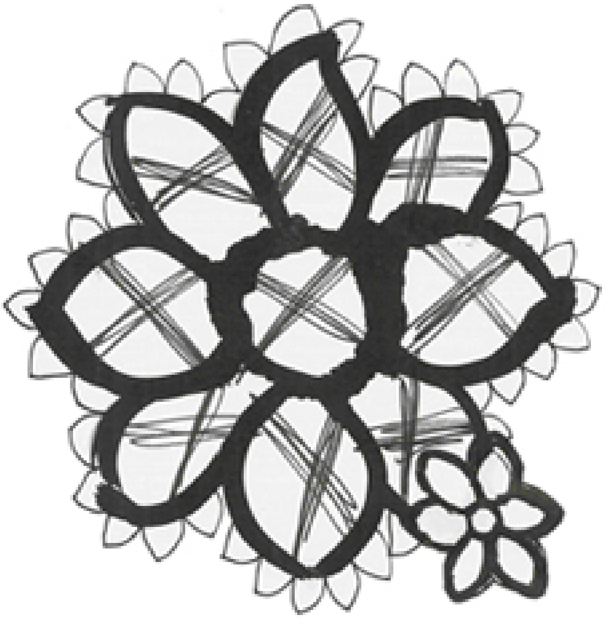

**FIGURE 1.** The ego's relationship to the life mission, or purpose in life. The talents supporting one's true purpose in life relate to one's true self, depicted here as the big flower, while the ego is the small flower that centers on a secondary or (as seen here) a tertiary purpose in life and the associated talents. The drawing is made by a tinnitus patient, a female artist who could not work because of her disease. In therapy, she came to a sudden realization of her personal life mission, and shortly after this breakthrough, her tinnitus disappeared.

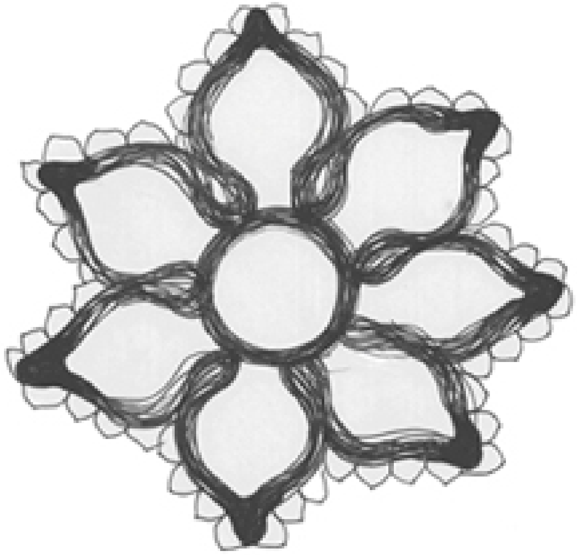

**FIGURE 2.** The structure of the life mission. This drawing is made by the same female tinnitus patient, and depicts the essence of her self as a flower of talents, with her life mission as the central core and the secondary talents as the petals; attached to these are tertiary talents and so forth. Two years after the therapeutic breakthrough that allowed her to formulate her life mission, her tinnitus seemed permanently healed and she is able to work again as an artist, more talented than before. The white flower has no dark petals (suppressed talents), illustrating that her ego has now been tuned to her life mission and her inner conflicts have lost their power to make her sick.

primary purpose in life (Fig. 2). This is because the original personality is still there, active and alive, behind the ego**.**

## THE PROCESS OF SALUTOGENESIS

Compared with the real self, the ego is weak. It seems that energy, joy in life, and intuitive competence pour from the true self, the primary purpose in life. The existential position far from our natural center of being is commonly known as the neurotic personality. Lacking much of the energy and the personal drive to overcome life's difficulties, the neurotic person often gives up, which explains depressions, feelings of insufficiency, low self-esteem, and the urge to commit suicide. Such phenomena, and many more, can be seen as symptoms caused by the loss of connection to the real self, the primary purpose in life.

Symptoms, disorders, and diseases that may be explained by the loss of energy, joy in life, and intuitive competence, may be alleviated or cured in the salutogenetic process[3] that helps patients find their primary purpose in life, their life mission. Many cases of reduced ability to function**,** physically as well as psychologically, socially and sexually, can also be explained in this way. When a person discovers their true talent and begins to use it with dedication, privately as well as professionally, his or her life will flourish and he or she may overcome even serious disease and great adversity in life[4,5,6]. The salutogenetic process can therefore also be called "life quality as medicine"[7,8].

It is important to notice that the plan for personal development laid out by this theory is a plan not for the elimination of the ego, but for its cultivation. An existentially sound person still has a mental ego of course, but it is centered on the optimal verbal expression of the life mission. Such an ego is not in conflict with one's true self, but supports the life and wholeness of the person, although in an invisible and seamless way. The more developed the person, the more talents are taken into use. So although the core of existence remains the same throughout life, the healthy person continues to grow. As the number of talents we can call on is unlimited, the journey ends only at death.

## TWO CASE STORIES

*A female, aged 42 years, with tinnitus, migraine, herpes simplex 1 and 2, low back pain, treatment-resistant genital warts, sun allergy, and depression. Despite her age, Mia (Figs. 1 and 2) was already in a very poor condition, physically and mentally. But she possessed something special, an alertness and interest in the spiritual world. She wanted to develop as a person and that meant that she was ready to assume responsibility and take the rather bitter, holistic medicine offered to her. We met in a good and sincere way. Processing her painful personal history took her directly to her life purpose. Following this acknowledgment, her art began to flourish and grow like never before. Suddenly, she could do things that she had not even come close to doing before, and her art expressed her new state of acceptance and understanding of good and evil, beautiful and ugly, muck and mire, and sky and light. Having acknowledged her life purpose, Mia largely became able to manage on her own. She could now develop further without our help. My work (SV) of guiding her through the pain that made her ill and blocked her enjoyment of life and self-expression is now finished. Her body and soul have largely healed, her tinnitus is almost gone, and most of the time she cannot hear it at all. Obviously, this patient may become physically ill again, but her resistance and inner equilibrium appeared to be much greater than before, so next time she is likely to recover much faster.*

*This woman seemed to have almost all her diseases caused by inner conflicts between her ego and her true self. When the conflicts were solved in holistic therapy, most of her seemingly incurable diseases disappeared at the same time.*

The next case story was written by a Rosen Body Work practitioner at the Quality of Life Research Center. It is instructive as it shows an important aspect of how the conflict of the ego vs. the true self is related to the subjective problems of a male with heart problems.

> *Male, aged 55 years, with the question if he has heart problems. This patient is a family man and manager in a private firm. He seems a happy and extroverted man with a good grip on things. However, his body was heavy and his muscles were very hard. Shortly before he started at the clinic of SV, he had been in hospital with a blood clot in his heart and was taking medication for hypertension.*
>
> *Most of the times he was on the couch, he fell into a deep sleep that was frequently interrupted by some very violent jerks throughout the body, which he called his electric shocks. Several times during the period when he came to see me, he was admitted to the hospital with extreme cardiac pain and angina. Eventually he started medication for these symptoms and was on the waiting list for bypass surgery. During some of his private sessions, he became aware of some of the things that had greatly influenced his life, including an alcoholic father who had been violent towards his mother. As a very young man, he received electroconvulsive therapy for severe depression.*
>
> *After he had realized this, the jerks that used to wake up both him and his wife ceased or diminished. It also became apparent to me that he was taking strong antidepressants and had done so for years. He now chose to reduce the dosage so that he was far below the daily dose, and he was doing well without the excessive medication.*
>
> *Throughout the therapy, he had some major problems with his staff and he felt they had taken a dislike to him. I had other clients from that workplace and it turned out that others shared his belief. The patient mobilized all his strength to give notice and start again from scratch in another firm, where he is working today.*
>
> *At some point, he was again admitted to the hospital with extreme pain and angina that was considered to be life threatening, so he was transferred to a cardiology ward for surgery at the earliest opportunity. However, when the cardiologists examined him thoroughly, they could not find any disorder or defect in the heart or surrounding blood vessels, so they discharged him again.*
>
> *During the last private session with the patient, he was truly happy about life, and full of vigor to devote to his family and friends. His jerks and cardiac problems had vanished completely, and he was enjoying his new job.*

What happened here according to the theory of the ego presented in this paper is that the man finally let go of his cold and frozen-hearted ego, which was suppressing his feelings and emotions, and which was very good for his subjective experience of his heart, his quality of life, working life, and ability to function in general.

The method of Marion Rosen Body Work and other body therapies make the patient note that the feelings located in the body are effective tools in holistic medicine. Sometimes the patient can verbalize feelings and let go of the limiting beliefs that keep them bound to the narrow world of the ego. For many middle-aged men, their Achilles' heal is allowing themselves to feel. Often, it is extremely unpleasant for a grown-up man in a managerial position to register the old feelings from his childhood of being small, frightened, or helpless. It is quite simply an insult to his ego that he is still harboring such feelings. To release them seemingly relieved his angina.

## DISCUSSION

The concept of the ego is only meaningful if one acknowledges the concept of a more real, more valuable, and more natural self. We know this “other self” indirectly, through the relationship with our self[9,10,11,12,13,14,15,16,17,18,19]. You can only have such a relationship with your self, if you have your “self” relating to the other. For most of us, the introspective scene is complicated, and not at all as clear as one could wish. So introspection is obviously the hard road to information about the human psyche.

In many respects, Freud and his brilliant student Jung[20] had been for the west what Buddha was for the east, enlightening us on the structure of the consciousness and subconsciousness of the human being. While Lord Guatama Buddha walked the hard road, starving himself almost to death, in his introspective search for the truth, Jung chose a completely different strategy, observing the other. This seems to be the western scientific approach to understanding — always watching, analyzing, and mapping the field through the other person. Interestingly, the elements of Jung’s map of human existence[20], being a pure product of his qualitative studies of human nature, is easy to identify for the therapist doing existential holistic therapy[21,22]. But as we all know, the perspective yields the vision. So to argue for the structure of the ego presented in this paper, you have to argue for the perspective. The perspective is the life mission theory[1], and it is very difficult to argue for this theory; it is really a matter of philosophical perspective on life and subsequent interpretation.

In the existential holistic therapy, we very often take people into deep regression into the womb and back to the beginning of life[23]. We do that because according to the life mission theory, we believe that life is whole from the very beginning, carrying our purpose and all the other qualities of consciousness, like joy and understanding. But this is a pure belief and this is confirming our own belief in the therapeutic process. This is not at all a solution to the famous hermeneutic problem[24]: That we, through all our efforts of investigation and reality testing, in the end only will confirm what we believed at the beginning — that is, if our worldview is consistent. A consistent view is not a true view. We are caught, as many philosophers have taught us, in our own description of the world. So knowing this, how can we argue that there is scientific truth in the mapping of the life purpose and the ego we propose in this paper?

Many people have doubted Jung and many more will doubt him in the future. We see this structure in our patients with no doubt at all because we share this perspective, but we must also admit that the structure of the ego is *a product* of a perspective of life that might in the end be very wrong, so why do we suggest this after all, and claim it to be science also?

The first argument is that, quite interestingly, the philosophy about the ego and the true self has been one of the conceptual keys, along with the concept of the evil, of most major medicine systems in most premodern cultures from Tibet, India, and Japan in the east to the African Sangoma’s, the Australian aboriginals in the south, the Shamans of the Eskimos of Greenland, the Same in the north of Scandinavia, to the native Americans in the west[25]. Realizing that all wisdom of all the premodern cultures can be brought together in one philosophical system made Aldous Huxley call his perhaps most famous and extraordinarily wise book on the human existential problems, *The Perennial Philosophy*[26]. In making such a synthesis of our own, much inspired by Huxley, we must agree to the critique that we tend to pull together psychoanalytic and existential thoughts. While these two perspectives have much in common, they are not the same regarding their view of human nature. We hope that the reader will forgive this unpleasant result of our eager attempt of simplification.

However convincing, the cultural argument is of course not a hard argument in medical science. In premodern time, most people believed in free spirits, reincarnation, and élan vital. They did not know the physiology of the heart or the structure of the brain. Knowing about neurobiology, mental projections, and neuropsychology, we do not see the spirits because we do not interpret reality that way, but still we definitely see the ego! So how can we argue for our perspective? Why is the ego in another category than the spirits? It definitely is as abstract and as difficult so see with the naked eye as the spirits.

The scientific exploration of the human existence in existential, holistic therapy seems to give us a consistent and well-structured worldview, that empowers us as holistic physicians to be powerful and supportive role-players in the patent’s “game of life”, which we see as the aim of holistic medicine. The scientific approach in holistic medicine is powerful, because it combines the consistency and structure of medical science with the good intention of supporting the very existence of the patient. Understanding existence, our own and our patient's, and meeting the patient with a good will — even with love — is in the end what makes us good physicians and excellent consultants.

When we understand human nature, we can help people to heal and support them to grow, because we perceive them as rich, beautiful, and gifted souls. In this way, we can be of value to our patients and clients — through this understanding of man, his ego, and his true self. This fruit of our combined academic and clinical efforts is what, in our understanding, makes it medical science or as we call it the "new medicine".

## CONCLUSION

As physicians or business consultants, we meet people who are feeling stuck in their lives, who are feeling empty or isolated, who are complaining about a poor quality of life, poor health, lack of joy, sexual interest, happiness, or talent. With the understanding of the nature of the human being in general and the person in front of us specifically, we should know precisely what to do, we should know how to help people grow and develop, and how to find hidden resources, talents, potentials, and possibilities. This can be done by helping them to move their existential position from the position of the ego, to the position of their real self, their life, their soul, and their wholeness.

Knowing the fundamental structure of the consciousness of man, we can often help the suffering person to heal his or her very existence. The dryness, the desolation, the stiffness, the stupidity, the emptiness, the righteousness, and the social isolation that characterizes people stuck in the position of the ego can be easily understood and made to go away, if the person cooperates. Even some physical or mental diseases can be healed by improving quality of life by such an approach[5,6,7,8,23].

So understanding makes us able to work holistically, while we focus on the whole person and not only the symptoms. Thus we can approach our patient and help him confront his imbalances and inner conflicts, and thus improve his state of being directly through his consciousness. When we succeed in this, we often do not need any drugs at all to help people get rid of pain, alcohol dependency, depression, anxiety, and other symptoms of poor quality of life[7,8].

The understanding of the ego and the real self seems to be one of the universal keys to see, meet, and help people to grow and heal in order to empower the physician or therapist to intervene successfully through the patient's consciousness. Antonovsky[3] saw this possibility clearly and called it salutogenesis — meaning something like "creation of solution". We believe that this difficult concept will have a great future in medical science. We use the simpler term "holistic healing" in the same sense[21,22]. It has nothing to do with energy-healing in the new age sense, but has everything to do with developing the consciousness, understanding, responsibility, and sense of coherence of our patient. Because the clever physician knows humbly that the healing powers are to be found within the patient, not within the physician, he is only the facilitator.

Understanding the concept of the ego as presented here, it is easy for the physician to motivate the patient to go through a lot of difficulties in order to grow and develop, and when the patient her- or himself fully understands the concept of the ego and the true self (higher self), the patient gets a strong feeling of direction in the personal development, and a motivation to fight the internal obstacles for quality of life, health, and the ability to function.

## BIOSKETCHES

**Søren Ventegodt, MD,** is the Director of the Quality of Life Research Center in Copenhagen, Denmark. He is also responsible for a Research Clinic for Holistic Medicine in Copenhagen and is a popular speaker throughout Scandinavia. He has published numerous scientific or popular articles and a number of books on holistic medicine, quality of life, and quality of working life. His most important scientific contributions are the comprehensive SEQOL questionnaire, the very short QoL5 questionnaire, the integrated QOL theory, the holistic process theory, the life mission theory, and the Danish Quality of Life Research Survey, 1991–94 in cooperation with the University Hospital of Copenhagen and the late pediatric professor Bengt Zachau-Christiansen. E-mail: ventegodt@livskvalitet.org. Website: www.livskvalitet.org/

**Niels Jørgen Andersen, MSc,** Professor, Department of Innovation and Economic Organization, Norwegian School of Management. This department conducts research and provides teaching in central topics related to innovation, business development, management of global companies, business history, and economic organization. Research activities within the Department are related to four core subjects within the discipline: business history, cooperative organizations, business development and entrepreneurship, and finally studies of industries with a special focus on the electricity industry. He is also the dynamic chairman of the nonprofit organization Stiftelsen Holistisk Medisin Scandinavia, which aims to support the scientific development, research, and documentation of complementary and holistic medicine in Scandinavia. E-mail: niels.j.andersen@bi.no. Website: www.bi.no/users/fgl93013/

**Joav Merrick, MD, DMSc,** is Professor of Child Health and Human Development affiliated with the Zusman Child Development Center, Division of Pediatrics and Community Health at the Ben Gurion University, Beer-Sheva, Israel; the Medical Director of the Division for Mental Retardation, Ministry of Social Affairs, Jerusalem; and the Founder and Director of the National Institute of Child Health and Human Development. He has numerous publications in the field of child and human development, rehabilitation, intellectual disability, disability, health, welfare, abuse, advocacy and prevention. Dr. Merrick received the Peter Sabroe Child Award for outstanding work on behalf of Danish Children in 1985 and the International LEGO-Prize ("The Children's Nobel Prize") for an extraordinary contribution towards improvement in child welfare and well being in 1987. E-mail: jmerrick@internet-zahav.net. Website: www.nichd-israel.com
